# Deciphering the Role of Extracellular Vesicles Derived from ZIKV-Infected hcMEC/D3 Cells on the Blood–Brain Barrier System

**DOI:** 10.3390/v13122363

**Published:** 2021-11-25

**Authors:** Antonios Fikatas, Jonas Dehairs, Sam Noppen, Jordi Doijen, Frank Vanderhoydonc, Eef Meyen, Johannes V. Swinnen, Christophe Pannecouque, Dominique Schols

**Affiliations:** 1 Laboratory of Virology and Chemotherapy, Department of Microbiology, Immunology and Transplantation, Rega Institute for Medical Research, Faculty of Medicine, KU Leuven, 3000 Leuven, Belgium; antonios.fikatas@kuleuven.be (A.F.); sam.noppen@kuleuven.be (S.N.); jordi.doijen@kuleuven.be (J.D.); eef.meyen@kuleuven.be (E.M.); christophe.pannecouque@kuleuven.be (C.P.); 2 Laboratory of Lipid Metabolism and Cancer, Department of Oncology, Faculty of Medicine, KU Leuven, 3000 Leuven, Belgium; jonas.dehairs@kuleuven.be (J.D.); frank.vanderhoydonc@kuleuven.be (F.V.); j.swinnen@kuleuven.be (J.V.S.)

**Keywords:** extracellular vesicles, Zika virus, blood–brain barrier, viral transmission, monolayer integrity, lipidomics

## Abstract

To date, no vaccines or antivirals are available against Zika virus (ZIKV). In addition, the mechanisms underlying ZIKV-associated pathogenesis of the central nervous system (CNS) are largely unexplored. Getting more insight into the cellular pathways that ZIKV recruits to facilitate infection of susceptible cells will be crucial for establishing an effective treatment strategy. In general, cells secrete a number of vesicles, known as extracellular vesicles (EVs), in response to viral infections. These EVs serve as intercellular communicators. Here, we investigated the role of EVs derived from ZIKV-infected human brain microvascular endothelial cells on the blood–brain barrier (BBB) system. We demonstrated that ZIKV-infected EVs (IEVs) can incorporate viral components, including ZIKV RNA, NS1, and E-protein, and further transfer them to several types of CNS cells. Using label-free impedance-based biosensing, we observed that ZIKV and IEVs can temporally disturb the monolayer integrity of BBB-mimicking cells, possibly by inducing structural rearrangements of the adherent protein VE-cadherin (immunofluorescence staining). Finally, differences in the lipidomic profile between EVs and their parental cells possibly suggest a preferential sorting mechanism of specific lipid species into the vesicles. To conclude, these data suggest that IEVs could be postulated as vehicles (Trojan horse) for ZIKV transmission via the BBB.

## 1. Introduction

Zika virus (ZIKV) is an emerging, single-stranded RNA virus of positive polarity that belongs to the large family of *Flaviviridae*. This flavivirus member is mainly transmitted to humans by two species of *Aedes* mosquitoes (*aegypti* and *albopictus*) [1]. In addition, several studies have described that ZIKV can be transmitted via other routes as well, including sexual contact as well as blood transfusion from mother to child [2,3]. ZIKV was first isolated in Uganda in 1947 from a febrile sentinel rhesus monkey. The first reported case of human infection with ZIKV occurred in 1962–1963, but was neglected until more recent outbreaks appeared on the Yap Islands (2007), French Polynesia (2013), and Brazil (2015) [4], after which the World Health Organization (WHO) declared the virus a Public Health Emergency of International Concern in 2016 [5]. As most infections caused by flavivirus members, ZIKV infection is usually asymptomatic with mild manifestations, such as headache, joint pain, and low-grade fever. However, several cases of ZIKV-associated neurological manifestations raised questions in the scientific community and encouraged them to study how this virus is able to cross physical barriers (e.g., placenta and blood–brain barrier), reaches the central and peripheral nervous system, and mainly infects the fetal brain and neural progenitor cells [6]. The permissiveness of these cells to ZIKV could possibly explain the neuronal abnormalities such as microcephaly [7,8] and Guillain-Barré syndrome [9,10]. Even though extensive efforts have been made to understand which factors lead to ZIKV-associated neuropathogenesis, our knowledge is still very limited. So far, no antivirals or vaccines are available to combat this virus.

In general, viruses infect and replicate into their hosts by hijacking a number of cellular mechanisms. Several studies have reported that viruses can exploit the exosomal pathway in order to spread their infection and further modify the function of target cells [11,12]. This particular pathway controls the intercellular communication via membrane-enclosed vesicles, known as extracellular vesicles (EVs). EVs consist of three main types of vesicles, depending on their biogenesis mechanisms [13]. Particularly, exosomes (50–150 nm) follow the endocytic route and are formed in the late endosomes (multivesicular bodies, MVBs). Fusion of MVBs with the plasma membrane leads to their secretion into extracellular space. On the other hand, microvesicles (100–1000 nm) and apoptotic bodies (500 nm–2 μm) are directly released from the plasma membrane [14]. Even though these distinct EV populations are secreted by all cell types, it is extremely challenging to properly discriminate them. For that reason, scientists suggest the use of small and medium/large extracellular vesicles (EVs) as nomenclature to describe EVs smaller and larger than 200 nm in size, respectively, according to the Minimal Information for Studies of Extracellular Vesicles (MISEV 2018) [15]. 

In order to study the role of EVs in viral infections, it is critical to isolate them and obtain pure vesicles. This is a challenging task, since viruses and EVs share multiple pathways and can be present at similar sites such as the plasma membrane and MVBs [16]. Particularly, in the case of ZIKV, the virus enters into cells through the clathrin-dependent pathway (primary route of EV uptake from target cells) and follows exocytosis during its budding and egress [17]. In addition, numerous studies have shown that several viruses are able to incorporate EV material in to their interior content and vice versa, thus making their precise discrimination more cumbersome [18,19].

Throughout literature, several reports describe the ability of ZIKV to reach into the CNS and infect susceptible cells including immature neural progenitors (but not mature neurons) [20], astrocytes [21], and microglia [22]. However, the mechanisms by which ZIKV crosses the blood–brain barrier (BBB), a very tightly sealed structure, are quite controversial. The BBB is mainly composed of brain microvascular endothelial cells surrounded by other cell types such as pericytes and microglia. It forms a dynamic and complex barrier that prevents microbes to pass from the peripheral circulation to the brain and CNS [23]. The fact that the BBB is highly semipermeable to substances raises many questions on the capability of ZIKV to pass through this system without being recognized by the host immune responses. 

In this study, we propose that EVs might serve as vectors to facilitate ZIKV transmission through the BBB. This hypothesis is based on the fact that EVs act as intercellular communicators by transferring proteins, lipids, and nucleic acid molecules to recipient cells [24,25]. As such, we aimed to unravel the potential role of EVs from ZIKV-infected human brain microvascular endothelial cells in viral dissemination. First, EVs were successfully isolated from a well-studied in vitro model of the BBB (human brain microvascular endothelial cells, hcMEC/D3) by combining several isolation procedures, in order to minimize the possibility of viral contamination in our preparations. Next, we characterized the fractions by nanoparticle tracking analysis (NTA), transmission electron microscopy (TEM), and Western blot. In addition, the functional role of non-infected (NIEVs) and ZIKV-infected EVs (IEVs) was determined by a series of in vitro cellular assays. Our results demonstrated that IEVs can incorporate and deliver viral components (ZIKV RNA as well as NS1 and E proteins) into susceptible cell lines. Real-time impedance measurements showed that ZIKV and IEVs induce temporal disturbances in the monolayer integrity of BBB-mimicking cells during the first minutes of inoculation, potentially by inducing a rearrangement of VE-cadherin, a protein with a unique role in BBB architecture. In addition, a comprehensive lipidomics analysis revealed a potential selective sorting mechanism of specific lipid classes into EVs. By comparing the lipidomic profile of cell lysates and EV preparations, we pinpointed the significance of these macro biomolecules to biogenesis and signaling processes. Finally, we identified differences in the relative abundance (%mol) of various lipid species between NIEVs and IEVs, thus advancing them as potential biomarkers during ZIKV infection. Here, we propose that ZIKV-infected EVs constitute an efficient mechanism of virus transmission to the BBB system, mainly through transient disruption of monolayer integrity. 

## 2. Materials and Methods

### 2.1. Cell Culture and Viral Infection

Human brain microvascular endothelial cells (hcMEC/D3) (ATCC) were used in this study. Specifically, 75 cm^2^ flasks were pre-coated with 0.1% gelatin for 1–2 h, followed by washing steps with PBS. Then, low-passage hcMEC/D3 were cultured in EndoGRO-MV Basal Medium (5% serum, Merck Millipore, Overijse, Belgium) supplemented with the essential growth factors (Supplement kit). The ZIKV clinical isolate PRVABC59 (ATCC), from the Asian lineage, was used to infect hcMEC/D3 cells at a multiplicity of infection (MOI) of 0.5 for 72 h. 

### 2.2. Isolation of Extracellular Vesicles (EVs)

HcMEC/D3 (3 × 10^5^ cells/well) were cultured in 6-well plates (Corning, Corning, NY, USA) in EndoGRO-MV culture medium for 24 h. The next day, the culture medium was replaced and cells were washed with PBS and further incubated with heat inactivated exosome-depleted serum (Exo-FBS, System Biosciences, Palo Alto, CA, USA) for an additional 3 h. Cells were exposed to culture medium (non-infected) and ZIKV PRVABC59 at MOI 0.5 (virus-infected) for 72 h. Subsequently, cell culture medium was collected in sterile 50 mL tubes (Greiner Bio-One, Vilvoorde, Belgium), and differential centrifugation steps (500× *g* for 10 min, followed by 2000× *g* for 15 min) were applied to remove dead cells and cellular debris. Then, the supernatant for each condition was placed into 15 mL Amicon filter units (10 KDa, Merck Millipore, Belgium) and centrifuged at 4000× *g* for 30 min. Next, the fraction that remained on the filter was further passed through a syringe (0.2 μm pore size, Corning, USA). Finally, 1 mL of the concentrated sample was placed at the top of a discontinuous Optiprep^TM^ density gradient (from top to bottom iodixanol solution: 2.5 mL of 5% and 10%, 2 mL of 20% and 40%) in 10.2 mL reseal tube (Thermo Fisher Scientific, Waltham, MA, USA), followed by ultracentrifugation (no brake) at 100,000× *g* for 18 h (Th641 rotor, Beckman Coulter, Brea, CA, USA). Six different fractions of EVs per each condition (1.5 mL each) were put into new tubes (diluted in PBS) and subsequently ultracentrifuged at 100,000× *g* for 3 h. The pellet from each fraction was collected, diluted in 300 μL PBS, and stored at −80 °C for further analysis.

### 2.3. Protein Isolation and Western Blot Analysis

Proteins from non-infected EVs (NIEVs), infected EVs (IEVs), as well as cell lysates (virus and cell controls) were extracted using Radio Immuno Precipitation Assay (RIPA) buffer (89900, Thermo Fisher Scientific) with freshly added protease and phosphatase cocktail inhibitor (1861281, Thermo Fisher). Particularly, 20 μL of each EV fraction were mixed with an equal volume of RIPA buffer for 15 min on ice, where after the mixture was further diluted in 2× Laemmli buffer (33401-1VL, Sigma Aldrich, St. Louis, MO, USA) and heated at 95 °C for 15 min. Then, 30 μL of each sample were loaded into sodium dodecyl sulfate (SDS) polyacrylamide 4–12% ready gels (Bio-Rad, Hercules, CA, USA) and electrotransferred to a nitrocellulose membrane using a trans-blot transfer pack system (Biorad). Milk Tris-Buffered Saline with 0.05% Tween20 (TBS-T) was used to block the membranes for 1 h. Blots were probed with the following primary antibodies overnight at 4 °C: rabbit anti-CD63 (CD63A-1, 190523-001) anti-ALIX (ALIX-1, 180312-005), anti-TSG101 (TSG101-1, 180312-006) (1/1000, System Biosciences), anti-calnexin (PA5-34665) (1/1000, Invitrogen), and mouse anti-NS1 (5123) (1/1000, Virostat, Westbrook, ME, USA), diluted in 5% milk TBS-T. Finally, membrane blots were washed three times with 0.05% TBS-T and incubated with secondary anti-rabbit (P0399) and anti-mouse (P0447) antibodies (Dako, 1/4000) in 5% milk TBS-T for 1 h at room temperature. Finally, the blots were visualized using the ChemiDoc MP imaging system (Bio-Rad).

### 2.4. Transmission Electron Microscopy (TEM)

Non-infected and infected EVs were fixed with 2% paraformaldehyde (PFA, Merck Millipore, Burlington, MA, USA) for 10 min at room temperature and 3 μL of the mixtures were placed on glow-discharged 300 mesh Cu-grids (Ted Pella, Redding, CA, USA). In order to increase the number of vesicles, each sample was deposited multiple times on the same grid, each time followed by a brief blotting step. Then, samples were negatively stained by placing the grids on a drop of 1% uranyl acetate for 1 min, after which they were blotted and allowed to dry. Finally, grids were introduced in a transmission electron microscope (JEOL JEM1400, Jeol Ltd., Tokyo, Japan) equipped with an EMSIS Quemesa 11Mpxl camera and imaged at an acceleration voltage of 80 kV.

### 2.5. Nanoparticle Tracking Analysis (NTA)

The concentration and size distribution of EVs in different fractions were determined using the Nanosight model NS300 (Malvern Instruments Company, Malvern, UK). The instrument was cleaned using PBS, and the temperature was maintained at 25 °C. In addition, the detection threshold was set at 4 and the screen gain at 10, in order to remove any background for the measurement. Nanoparticles of 100 nm were used to calibrate the system prior to examination of our samples. Then, 1 mL of each fraction (diluted in PBS) was put into the syringe and slowly pushed through the laser module. Six recording video measurements (30 s each) were obtained for each EV sample. The data were averaged and analyzed using Nanosight NTA 2.3 software (Malvern, UK).

### 2.6. Immunofluorescence Staining and qPCR Analysis

Susceptible HcMEC/D3 (3 × 10^4^ cells/well) were seeded in gelatin pre-coated black 96-well plates (Falcon, Munich, Germany) for 96 h to achieve a confluent monolayer. Next, the cells were exposed to ZIKV *PRVABC59* (MOI 0.5) and different fractions of NIEVs/IEVs (100 μL) for 72 h. The amount of EVs, added during the assay, was normalized based on the number of particles (1.5 × 10^7^ particles). EV-depleted supernatant (supernatant taken after the second ultracentrifugation step) was used as negative control in the study. Cells were first fixed for 5 min with 2% paraformaldehyde (PFA) at room temperature and then permeabilized with 0.1% Triton X-100 (Sigma Aldrich) for 10 min. Later, 1% Bovine Serum Albumin (BSA, Cell Signaling Technology, Leiden, The Netherlands) was used to block the fixed cells for 1 h followed by two washing steps with PBS. Incubation of hcMEC/D3 cells with the primary 4G2 pan-flavivirus antibody (clone D1-4G2-4-15, Merck Millipore, Belgium) was performed overnight at 4 °C. Goat anti-mouse IgG Alexa Fluor 488 (Invitrogen, Waltham, MA. USA) was used as a secondary antibody. The nucleus was stained with DAPI (Invitrogen). Fluorescence microscopy was performed on a Zeiss Axiovert 200M inverted microscope (Zeiss, Göttingen, Germany) using Long Distance (LD) Plan-Neofluar 20×/0.4 objective.

For the detection of viral RNA in the vesicles, uninfected hcMEC/D3 were cultured in 96-well plates (Corning, USA) as previously described. ZIKV *PRVABC59*, IEVs, and NIEVs were added to the cells. Cell lysates were collected at 72 h post infection (p.i.) and viral RNA was measured by Cells Direct One-step qRT-PCR kit (Thermo Fisher), according to manufacturer’s instructions. RT-PCR was performed using the ABI 7500 Fast Real-Time PCR System (Applied Biosystems, NJ, USA). The primer and probe sequences used for qRT-PCR were: 5′-TGA CTC CCC TCG TAG ACT G-3′ (ZIKV forward), 5′-CAC CTT TAG TCA CCT TCC TCT C-3′ (ZIKV reverse) and 5′-6-FAM/AGA TCC CAC/ZEN/AAA TCC CCT CTT CCC/3IABkFQ-3′ (ZIKV probe). The following amplification conditions were used for qRT-PCR: 50 °C for 15 min, 95 °C for 2 min, followed by 45 cycles of 95 °C for 15 s and 60 °C for 1 min. The infection efficiency was calculated using a curve of serial dilutions of ZIKV standard sample. All data were analyzed in GraphPad Prism 7 software (San Diego, CA, USA).

### 2.7. Transwell Assay

HcMEC/D3 (3 × 10^4^ cells) were seeded on 24-well transparent filter inserts (0.4 μm) (Greiner Bio-One) for 4 days. As soon as hcMEC/D3 cells formed a confluent monolayer (microscopic and impedance-based biosensing evaluation), both ZIKV *PRVABC59* (virus stock, MOI 0.5) and EVs (1.5 × 10^7^ particles) were added to the upper chamber of the insert. Human primary glioblastoma U87-MG (10^4^ cells) was also added to the bottom of the lower chamber. Cell lysates from U87-MG were collected 6 days post-infection and viral RNA levels were measured by qPCR, as previously described.

### 2.8. Label-Free Impedance Based Biosensor Analysis 

8-well 20idf-PET plates (Applied Biophysics, New York, NY, USA) were coated with 0.1% gelatin for 1 h and washed with MilliQ. Then, 100 μL of growth medium (EndoGRO-MV Basal Medium) were added to each well and the plate was placed in the 16-well module of the Electric Cell-substrate Impedance Sensing (ECIS) device (Applied Biophysics) that was located in an incubator (37 °C, 5% CO_2_). The 8WCP array type was chosen in the setup, and the plate was stabilized using the built-in stabilization function. After removing the plate from the device, 200 μL of hcMEC/D3 cells were added to achieve a density of 3 × 10^4^ cells/well. Plates were then left on the benchtop for 30 min to let the cells settle to the bottom. Next, the plate was placed in the ECIS device for 4 days to achieve confluency. The growth of the cells was continuously monitored using the multifrequency mode (MFT), thereby obtaining frequency measurements at 4, 16, and 64 kHz. Before addition of the samples, the measurement was paused. Then, 100 μL of either NIEVs, IEVs (same number of particles) or virus (MOI 0.5) were added to the cells, and the MFT measurement was resumed for up to 50 h with a time interval of approximately 3.5 min. Data were exported as abp files and further analyzed using the ECIS software (Troy, NY, USA). Both the resistance (obtained at low frequency current—4000 Hz) and capacitance (obtained at high frequency current—64,000 Hz) were used to assess whether ZIKV and EVs could induce cellular changes. The data were normalized by dividing each data point of a well by the value of that well at the time point prior to sample addition.

### 2.9. Immunofluorescence Staining and Western Blot Analysis for VE-Cadherin Detection 

HcMEC/D3 (3 × 10^4^ cells/well) were seeded as previously described. ZIKV *PRVABC59*, IEVs, NIEVs, and TNF-α (100 ng/mL) were added to the cells for 30 min, 1 h, and 24 h. After fixation, permeabilization, and blocking steps, cells were incubated with the primary CD144 (VE-cadherin) monoclonal antibody (clone 14-1449-82, Invitrogen) overnight at 4 °C. Goat anti-mouse IgG Alexa Fluor-488 was used as a secondary antibody, and the nucleus was stained with DAPI. Fluorescence microscopy was performed using LD Plan-Neofluer 40×/0.6 objective. For the Western blot analysis, cell lysates were collected at 30 min, 1 h, and 24 h p.i. and proteins were further extracted by a solution containing RIPA buffer along with the cocktail phosphatase/protease inhibitor. Fifteen micrograms per milliliter of lysate per condition (30 μL) were loaded into a SDS polyacrylamide gel and transferred to a membrane as mentioned above in detail. Mouse anti-CD144 (1/500) was used to probe the membranes (overnight), followed by incubation with the secondary anti-mouse antibody for 1 h, before the visualization of the blots.

### 2.10. Statistical Analysis 

Two different statistical tests were implemented to evaluate the significance of our results. Particularly, unpaired *t*-test was performed for Figure panels 1B, 2C, and 5B, while one-way ANOVA (Tukey’s post-correction test) was used to perform multiple comparisons in Figure panels 2B, 3B, and 4B. Significance was calculated based on *p*-values. ns: *p* > 0.05, * *p* < 0.05, ** *p* < 0.005, *** *p* < 0.0005, **** *p* < 0.0001

### 2.11. Lipidomic Analysis 

#### 2.11.1. Lipid Extraction 

Each sample (700 μL) was mixed with 800 μL 1 N HCl:CH_3_OH 1:8 (*v*/*v*), 900 μL CHCl_3_, 200 μg/mL of the antioxidant 2,6-di-tert-butyl-4-methylphenol (BHT; Sigma Aldrich), and 3 μL of SPLASH^®^ LIPIDOMIX^®^ Mass Spec Standard (#330707, Avanti Polar Lipids, Alabaster, AL, USA). After vortexing and centrifugation, the organic fraction was evaporated using a Savant Speedvac spd111v (Thermo Fisher Scientific) at room temperature and the remaining lipid pellet was stored at—20 °C under argon atmosphere.

#### 2.11.2. Mass Spectrometry

Just before mass spectrometry analysis, lipid pellets were reconstituted in 100% ethanol. Lipid species were analyzed using liquid chromatography electrospray ionization tandem mass spectrometry (LC-ESI/MS/MS) on a Nexera X2 UHPLC system (Shimadzu, Kyoto, Japan) coupled with hybrid triple quadrupole/linear ion trap mass spectrometer (6500+ QTRAP system; AB SCIEX). Chromatographic separation was performed on a XBridge amide column (150 mm × 4.6 mm, 3.5 μm; Waters, Milford, MA, USA) maintained at 35 °C using mobile phase A [1 mM ammonium acetate in water-acetonitrile 5:95 (*v*/*v*)] and mobile phase B [1 mM ammonium acetate in water-acetonitrile 50:50 (*v*/*v*)] in the following gradient: (0–6 min: 0% B → 6% B; 6–10 min: 6% B → 25% B; 10–11 min: 25% B → 98% B; 11–13 min: 98% B → 100% B; 13–19 min: 100% B; 19–24 min: 0% B) at a flow rate of 0.7 mL/min, which was increased to 1.5 mL/min from 13 min onwards. SM, CE, CER, DCER, HexCER, and LacCER were measured in positive ion mode with a precursor scan of 184.1, 369.4, 264.4, 266.4, 264.4, and 264.4 respectively. TG, DG, and MG were measured in positive ion mode with a neutral loss scan for one of the fatty acyl moieties. PC, LPC, PE, LPE, PG, LPG, PI, LPI, PS, and LPS were measured in negative ion mode with a neutral loss scan for the fatty acyl moieties. Lipid quantification was performed by scheduled multiple reactions monitoring (MRM), the transitions being based on the neutral losses or the typical product ions as described above. The instrument parameters were as follows: Curtain Gas = 35 psi; Collision Gas = 8 a.u. (medium); IonSpray Voltage = 5500 V and −4500 V; Temperature = 550 °C; Ion Source Gas 1 = 50 psi; Ion Source Gas 2 = 60 psi; Declustering Potential = 60 V and −80 V; Entrance Potential = 10 V and −10 V; Collision Cell Exit Potential = 15 V and −15 V.

The following fatty acyl moieties were taken into account for the lipidomic analysis: 14:0, 14:1, 16:0, 16:1, 16:2, 18:0, 18:1, 18:2, 18:3, 20:0, 20:1, 20:2, 20:3, 20:4, 20:5, 22:0, 22:1, 22:2, 22:4, 22:5, and 22:6, except for TGs, which considered: 16:0, 16:1, 18:0, 18:1, 18:2, 18:3, 20:3, 20:4, 20:5, 22:2, 22:3, 22:4, 22:5, and 22:6.

#### 2.11.3. Data Analysis

Peak integration was performed with the MultiQuant^TM^ software version 3.0.3 (Framingham, MA, USA). Lipid species signals were corrected for isotopic contributions (calculated with Python Molmass 2019.1.1) and were normalized to internal standard signals. Unpaired *t*-test *p*-values and False Discovery Rate corrected *p*-values (using the Benjamini/Hochberg procedure) were calculated in Python StatsModels version 0.10.1.

#### 2.11.4. EV-TRACK ID

Finally, we submitted all relevant data of our experiments to the EV-TRACK knowledgebase (EV-TRACK ID: EV210043) (Van Deun J, et al. EV-TRACK: transparent reporting and centralizing knowledge in extracellular vesicle research. Nature methods. 2017;14(3):228–232).

## 3. Results

**Figure 1:**
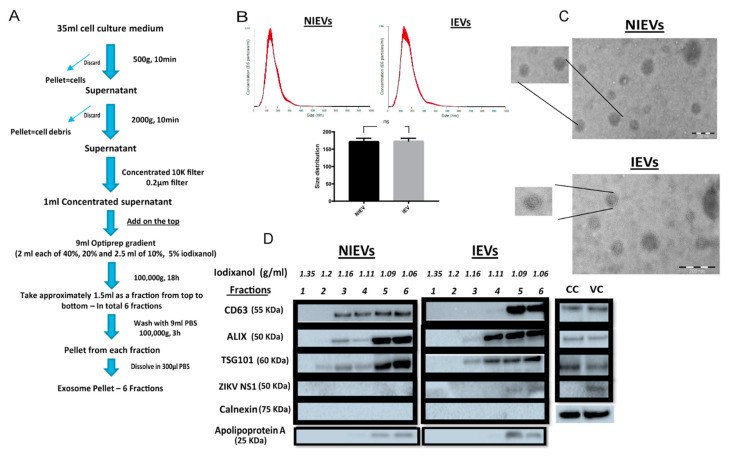
Isolation and characterization of non-infected and ZIKV-infected cell-derived EVs. (**A**) Experimental workflow for extracellular vesicle (EV) isolation from non-infected/ZIKV-infected human brain microvascular endothelial cells (hcMEC/D3). (**B**) Nanoparticle tracking analysis reveals no significant differences in size and number of particles between non-infected (NIEVs) and ZIKV-infected EVs (IEVs). (**C**) Electron microscopy indicates the presence of membrane-enclosed vesicles smaller than 200 nm in both non-infected (upper) and infected (bottom) EV preparations. Scale bar graph: 200 nm. (**D**) Western blot analysis demonstrates the presence of distinct profiles of several EV- and non-EV markers among fractions of both NIEVs and IEVs. Thirty microliters of each fraction (density is indicated in g/mL) were loaded into the SDS polyacrylamide gel. Lysates from non-infected (CC) and ZIKV-infected cells (VC) were also included.

Here, we used hcMEC/D3 cells, a stable and well-characterized model of human brain microvascular endothelial cells that maintains the BBB phenotype in vitro. In order to efficiently infect these cells, three different ZIKV strains were used (ZIKV *MR766*—African lineage, ZIKV *FLR* (Columbia) and ZIKV *PRVABC59* (Puerto Rico)—Asian lineage) at multiplicity of infection (MOI) of 0.5, 1, and 5 for 48 h and 72 post infection (data not shown). Our analysis showed that inoculation of hcMEC/D3 cells with ZIKV *PRVABC59* at an MOI of 0.5 presented a high infection rate with no cytotoxicity levels at 72 h p.i, as compared to these observed for other viral strains at different MOIs. HcMEC/D3 cell-derived EVs from infected and non-infected condition were isolated using a well-established protocol described by Zhou *W* et al. [26] with some slight modifications (Figure 1A). Particularly, differential centrifugation, followed by (ultra)filtration and density gradient separation were applied in order to acquire our EV preparations. In order to proceed with the characterization of our vesicles and the assessment of their functionality, we divided the EVs into six different fractions. The density of each fraction (1.06–1.35 g/mL) was determined by a control density gradient experiment using similar volumes of each iodixanol solution, as indicated in the Materials and Methods section. Cell viability was also assessed in parallel and measured at 95% throughout all experiments. Several conventional methods were performed to characterize our EV populations. Specifically, NTA analysis revealed that the presence of a virus did not significantly change the size of infected EVs (IEVs), as compared to the control ones (NIEVs) (approximately 170 nm) (Figure 1B). In addition, the number of particles did not seem to significantly change between the two conditions (approximately 1.5 × 10^8^ particles/mL). Furthermore, we examined the presence of EVs by TEM. As shown in Figure 1C, we were able to detect “cup-shaped” vesicles, smaller than 200 nm in both NIEVs (upper) and IEVs (bottom). Although we did not observe any viral particles in our EM images, derived from IEV preparations, we cannot exclude the possibility of their presence in these fractions. Finally, Western blot analysis was used to evaluate the presence of specific EV markers (Figure 1D). Interestingly, differences in the presence of certain markers were observed not only between the NIEVs and IEVs, but also among the different fractions. Notably, the tetraspanin CD63 was detected in fractions 3, 4, 5, and 6 of both NIEVs and IEVs, suggesting the presence of EVs. However, there was a clear increase in CD63 levels in fractions 5 and 6, as compared to the amounts in fractions 3 and 4 of IEVs. Similar results were noticed for ALIX and TSG101, two proteins with a crucial role in EV biogenesis, in fractions 3, 5, and 6 between NIEVs and IEVs. However, an increase in expression levels for both markers was detected in fraction 4 of IEVs, as compared to this of NIEVs. To define the purity of our EVs, we evaluated whether calnexin and apolipoprotein A were present in the fractions. Calnexin is an endoplasmic reticulum protein that is often used as a negative marker in most EV studies. As expected, calnexin was only detectable in cell lysates (CC: non-infected and VC: ZIKV-infected cells). On the other hand, apolipoprotein A is used to indicate the presence of high-density lipoproteins (HDL), since they can co-isolate with EVs due to their similar density. Interestingly, apolipoprotein A was mainly detected in the upper fractions (5 and 6) in both EV populations, whereas lower levels were noticed in fraction 4. Finally, we examined whether IEVs were able to incorporate any viral proteins into their content. In particular, ZIKV NS1 protein was solely detected in the upper fractions of IEVs. On the other hand, no NS1 was detected in any of the NIEV fractions nor in the cell control (lysate). These data suggest that our EVs are highly heterogeneous in size and composition and can incorporate viral components in their interior. 

**Figure 2:**
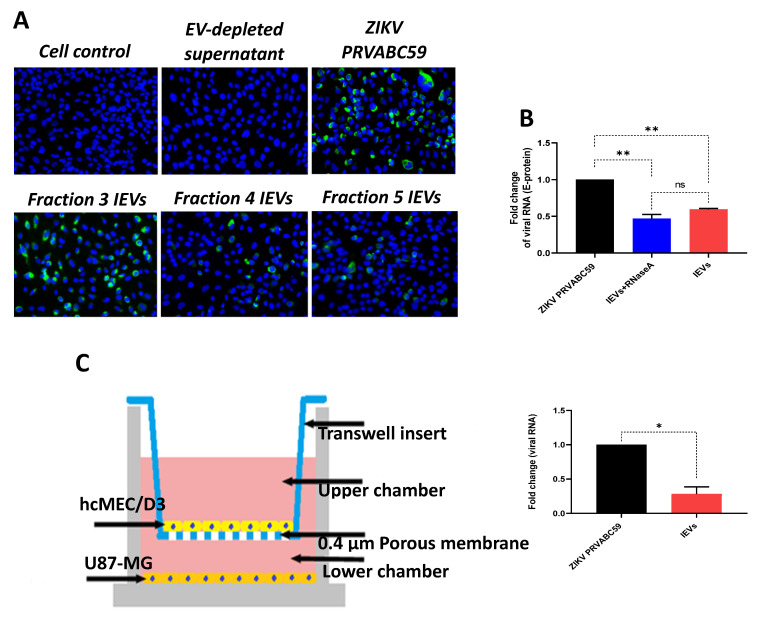
ZIKV-infected cell-derived EVs (IEVs) are able to transfer viral material to naïve cells. (**A**) Detection of ZIKV envelope protein (green) in hcMEC/D3 cells after their inoculation (72 h) with different IEV fractions (20× objective). Positive (ZIKV-infected cells) and negative (EV-depleted supernatant) controls are also included. (**B**) Fold change in viral RNA levels (detection of E-protein) among ZIKV- (black), IEVs-pretreated with RNase A (blue), and IEVs- (red) infected hcMEC/D3 cells. Pretreatment of IEVs with RNase A shows no differences in viral RNA levels with untreated IEVs, indicating that most of the RNA resides inside the vesicles. (**C**) Representation of the Transwell assay setup to demonstrate that IEVs (colored spheres), isolated from ZIKV-infected hcMEC/D3 cells, can transfer viral RNA to glioblastoma cells (U87-MG, lower chamber) (6 days post-infection). ZIKV virions (virus stock) was also included in the assay. Significant difference is also indicated between ZIKV- and IEV-treated cells and calculated based on *p*-values; ns: *p* > 0.05, * *p* < 0.05, ** *p* < 0.005. Data are acquired from two independent experiments.

Since we observed that secreted NS1 protein was detectable in some IEV fractions, we examined whether these vesicles were able to transfer other viral material to naïve cells. Interestingly, viral envelope glycoprotein (E protein) was detected in cells exposed to several IEV fractions (Figure 2A). However, the amount of E protein was different among the tested fractions. EV-depleted supernatant (supernatant obtained during the second round of ultracentrifugation, during which EVs were acquired from the pellet) was used as negative control, while virus-infected cells as positive. In addition, we determined whether ZIKV RNA could be detected in IEVs-infected cells, using quantitative real-time PCR (E-protein detection). As shown in Figure 2B, incubation of uninfected hcMEC/D3 cells with IEVs resulted in the detection of ZIKV RNA. In parallel, inoculation of cells with ZIKV *PRVABC59* strain led to higher levels of viral RNA with a significant difference between ZIKV- and IEVs-infected cells. To rule out the possibility of RNA being present outside of our vesicles, we pretreated IEVs with RNase A (5 μg/mL) for 15 min, followed by their incubation in recipient hcMEC/D3 cells. As can be seen in Figure 2B, no significant differences were observed in the viral load between the RNase A-treated and non-treated IEVs. Since the BBB is a complex structure and it is not only composed of microvascular endothelial cells, we aimed to mimic this system in vitro using a transwell assay. In this experiment, U87-MG glioblastoma cells were seeded at the bottom chamber, while hcMEC/D3 were placed at the upper one. After achieving confluence, the hcMEC/D3 monolayer was exposed to IEVs. These vesicles were able to pass through the monolayer and further deliver viral RNA to U87-MG cells (Figure 2C). ZIKV virions (virus stock) were also added on hcMEC/D3 cells as a positive control in this assay (significant difference was observed between the two conditions). 

**Figure 3:**
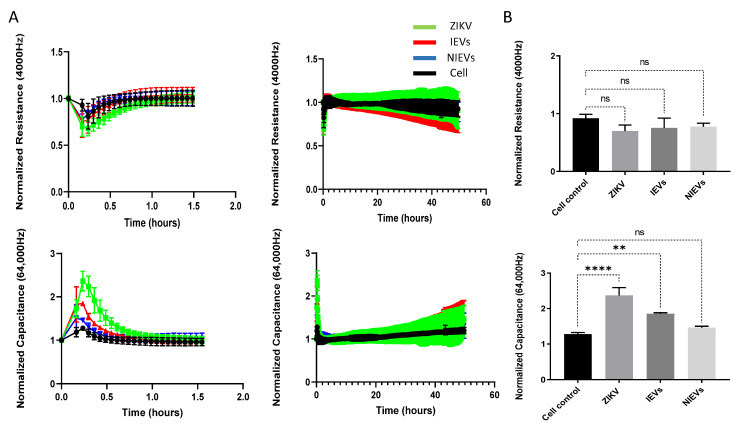
Both ZIKV and IEVs induce temporal disturbances in the monolayer integrity of BBB during the first minutes. (**A**) Normalized resistance (4000 Hz, **upper**) and capacitance (64,000 Hz, **bottom**) values indicate that ZIKV (green) and IEVs (red) can induce temporal changes in monolayer integrity of hcMEC/D3 cells, which could potentially result in passing via transcellular route within the first 30–45 min (**left panel**). However, no further changes are observed during the later stages (2 days post-infection, **right panel**). (**B**) Maximal changes (as compared to cell control, black) in the permeability of BBB during the first minutes demonstrate that ZIKV and IEVs (not NIEVs, blue) significantly alter capacitance (**lower**), but not resistance (**upper**) values. Normalization of the resistance/capacitance values was performed by dividing each data point by the value at the time point prior to sample addition. Data are acquired from three independent experiments. Significance is calculated based on *p*-values. ns: *p* > 0.05, * *p* < 0.05, ** *p* < 0.005, *** *p* < 0.0005, **** *p* < 0.0001.

**Figure 4:**
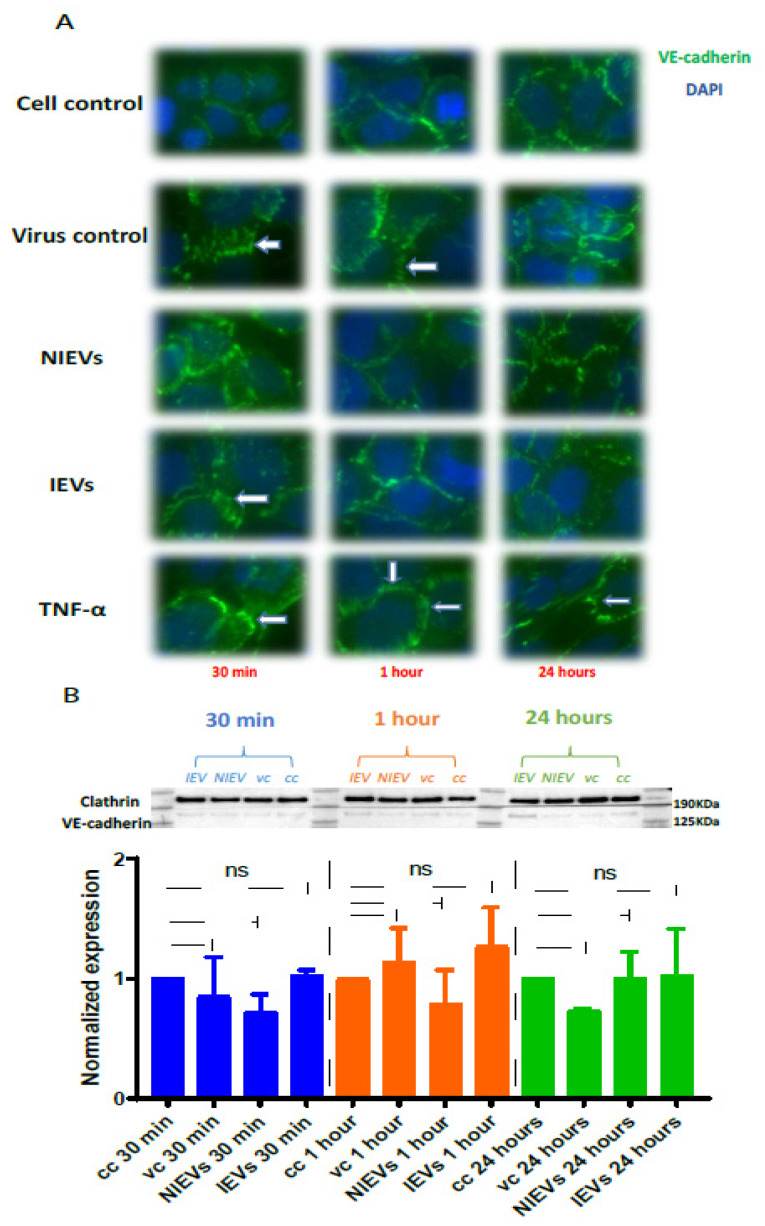
ZIKV and IEVs induce structural rearrangements of VE-cadherin at the early time points. (**A**) Alterations in the architecture of VE-cadherin (white arrows) are observed at 30 min in ZIKV-infected and IEV-treated hcMEC/D3 cells. These changes are restored at later time points, except for ZIKV-infected cells, where reorganization of VE- cadherin is still detectable at 1 h post infection. TNF-α (100 ng/mL) is used as a positive control (40× objective). (**B**) Expression levels of VE-cadherin are not significantly changed in ZIKV- and EV-treated cells after 30 min, 1 h, and 24 h. Clathrin is used as an internal loading control in the Western blot analysis.

Next, we evaluated the potential mechanisms that both virus and EVs use to pass through the BBB system, by applying a real-time electrical impedance assay. As shown in Figure 3, cellular changes were measured at different frequencies. Specifically, low frequencies (4000 Hz, Resistance) give an estimation of cell–cell contacts (paracellular route), while higher frequencies (64,000 Hz, Capacitance) demonstrate how confluent the monolayer is (transcellular route) [27]. After the addition of ZIKV and IEVs, we observed significant changes in capacitance, during the first 30–45 min of incubation, but not at later stages (2 days p.i.) (Figure 3A). More specifically, a subtle, non-significant decrease in resistance was observed after ZIKV/EVs addition, as compared to the mock-infected condition (cell control) (*p* > 0.05) (Figure 3B). Looking at high frequencies (64,000 Hz), ZIKV and IEVs (not NIEVs) significantly increased the capacitance value, suggesting that they induce temporary changes in the monolayer integrity (Figure 3B). These transient disturbances in the monolayer of the BBB model were further investigated by evaluating the expression levels of VE-cadherin, an adherent protein exclusively found in endothelial cells with a unique role in maintaining their barrier integrity. Particularly, immunofluorescence staining and Western blot analysis were performed at three different time points (30 min, 1 h, and 24 h p.i.). As shown in Figure 4A (immunofluorescence), incubation of hcMEC/D3 cells with ZIKV and IEVs resulted in changes in the architecture of VE-cadherin at 30 min p.i. (indicated by white arrows). However, this observation was less pronounced when the cells were inoculated with the NIEVs. These subtle modifications seem to be restored at later stages, except for ZIKV, where blunt changes were still observed at 1 h p.i. TNF-α was also used as a positive control in this study, since it has been associated with the dysregulation of VE-cadherin. In addition, Western blot analysis revealed that the expression levels of VE-cadherin did not significantly change among different time points after the addition of ZIKV and EVs, as compared to cell control (Figure 4B). 

**Figure 5:**
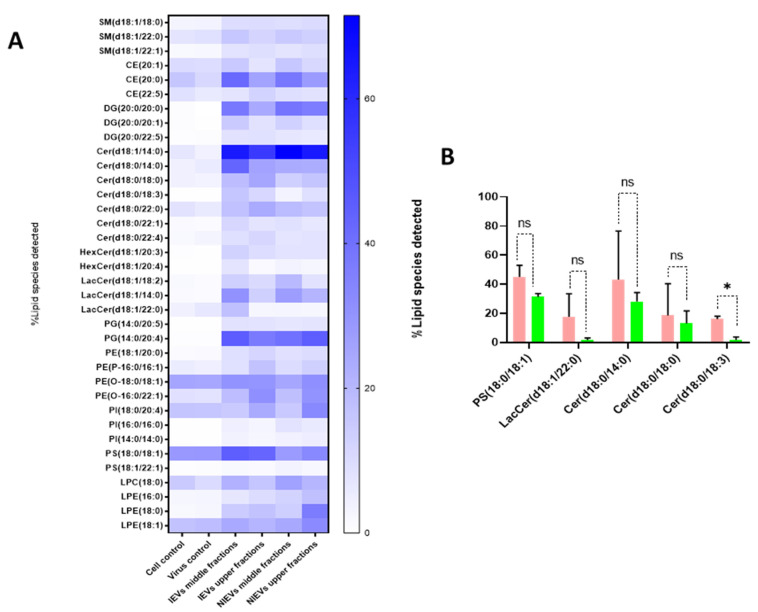
Distinct differences in the lipidomic profile between cell lysates and EV preparations, as well as NIEVs and IEVs. (**A**) Lipidomic heat map showing an increase (in percentage) of molecular lipid species per class into EV preparations, as compared to cell lysates. Each horizontal row represents a molecular lipid and each vertical column represents a sample. Lipid abundance ratios are colored according to their percentage. Colored boxes are used to compare the abundance of lipid species within each different class. (**B**) Five distinct lipid species have shown to be upregulated in infected condition (%mol), but only the levels of Cer(d18:0/18:3) are significantly changed in IEVs (pink), as compared to NIEVs (green) (*p* < 0.05). SM: sphingomyelin, CE: cholesteryl ester, DG: diacylglycerol, Cer: ceramide, LacCer: lactosylceramide, HexCer: hexosylceramide, PG: phosphatidylglycerol, PE: phosphatidylethanolamine, PI: phosphatidylinositol, PS: glycerophosphoserine, LPC: lysophosphatidylcholine, LPE: lysophosphatidylethanolamine. Data shown are from 3 independent experiments (mean ± SD values). Significance is calculated based on *p*-values. ns: *p* > 0.05, * *p* < 0.05.

As shown, IEVs can be used by ZIKV as vehicles to deliver their infectious content to recipient cells. Besides proteins and viral RNA, these vesicles have a high lipid content, which plays an essential role in EV biogenesis and signal transduction. As such, we aimed to characterize the lipidomic profile of hcMEC/D3 cells and EVs. As indicated by the Western blot analysis (Figure 1), apoA was detectable in the upper fractions (5 and 6), but not in the middle (3, 4) and lower ones (1, 2). Hence, we divided our EVs into two different groups (upper and middle fractions) for analysis, based on the levels of apolipoprotein A, which possibly indicates the co-presence of HDL in our preparations. A total of approximately 2000 lipid species from 14 different lipid classes were identified by a well-established lipidomic analysis (SM, CE, Cer, LacCer, HexCer, PC, LPC, PE, LPE, PG, PI, PS, TG, and DG). The data are shown as percentage of the total amount of lipid species per class (%mol) (Figure 5 and Figure S1). The most abundant lipid species/class (~200) were illustrated as heatmaps in Figure 5 as well as in Figure S1. Consistent with other reports on lipid content of EVs [28], we observed that our vesicles are mainly enriched in cholesterol, SM, and DG, as compared to most of the phospholipids and especially PC. Focusing on each lipid class separately (colored boxes to group and compare the abundance of lipid species within each class), a number of lipid species were found to be largely present in both cells and EVs, indicating their preference in the sorting mechanism. Specifically, LPE(20:4), LPC(18:1), SM(18:1/16:0), and LacCer(18:1/16:0) were the most abundant lipid species of their class. On the other hand, PE(18:1/18:1), LPC(18:1), PG(16:0/18:1), PI(18:0/20:3), PC(P-18:0/18:1), and HexCer(18:1/16:0) levels were much higher in cells, as compared to those in EV preparations (Figure S1). Finally, all lipid species/class that were enriched in EVs were depicted in Figure 5A. The most pronounced differences were noticed for Cer(18:1/14:0), DG(20:0/20:0), and PG(14:0/20:4), three lipids with a crucial role in the activation of signaling cascades and the maintenance of membrane curvature. Furthermore, it is noteworthy to mention the differences among the tested groups, as slight changes (in the percentage) were observed between the middle (absence of apoA) and upper (presence of apoA) fractions, indicating that lipoproteins can affect the way we interpret the results. Finally, Cer(18:0/18:3) was significantly increased in IEVs, as compared to the NIEVs (relative distribution), while four other lipid species, including sphingolipids (Cer and LacCer) and PS(18:0/18:1), also appeared slightly increased (non-significant) in the infected condition as compared to the non-infected one (Figure 5B). These findings suggest the importance of lipid molecules, as potential “signatures” during ZIKV infection.

**Figure 6:**
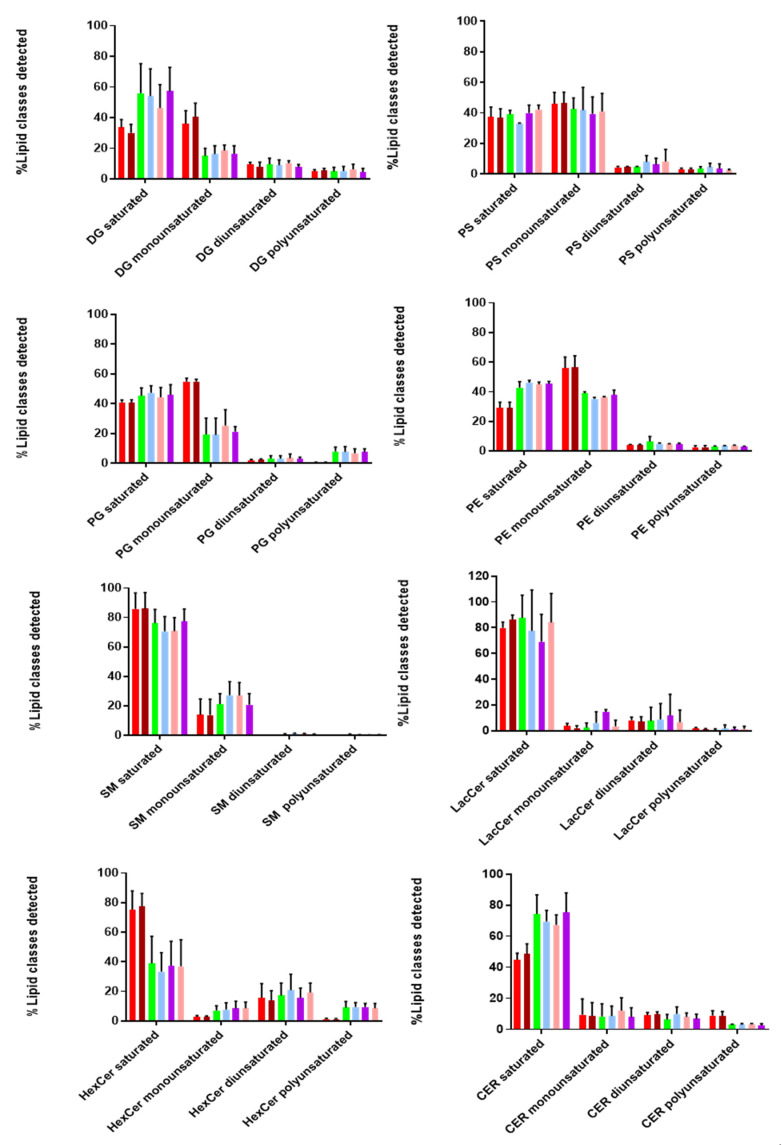
Degree of saturation of lipids in EVs and cells. Most of the detected lipid classes are saturated or monounsatutared, as compared to di- and poly-unsaturated ones in both EV preparations and cell lysates. Different color bars are depicted: Cell control (red), Virus control (brown), NIEVs middle fractions (green), NIEVs upper fractions (light blue), IEV middle fractions (purple), IEVs upper fractions (pink). DG: diacylglycerol, PS: glycerophosphoserine, PE: phosphatidylethanolamine, PG: phosphatidylglycerol, SM: sphingomyelin, LacCer: lactosylceramide, HexCer: hexosylceramide, Cer: ceramide. Data shown are from three independent experiments. The results are expressed as molar percentages (mol%).

Additionally, we looked at the degree of saturation (%mol) of the detected lipid classes, as it provides information on the structure and functionality of these biomolecules. As shown in Figure 6, both EVs and cell lysates contain more saturated and monounsaturated fatty acids compared to di- and poly-unsaturated ones. Taking a closer look at the lipid classes found in the inner and outer leaflet, we found that SM, Cer, LacCer, and HexCer (outer) contained more saturated and less monounsaturated fatty acids in both EVs and cell lysates. On the other hand, lipid classes found in the inner leaflet (PS, PE, PG) consisted of similar levels of saturated and monounsaturated fatty acids, while no diunsaturated lipids were detected in any of these lipid classes, except for HexCer and LacCer (~20%). Polyunsaturated fatty acids were not observed for SM, while higher levels were detected for HexCer. Moreover, it is interesting to point out that DG and Cer contain more saturated fatty acids in EVs than cell lysates. Finally, we observed a similar tendency in the degree of saturation for all tested lipid classes in both EVs and cell lysates. These notable differences in the degree of saturation can affect membrane dynamics and stability of EVs [28].

## 4. Discussion

Although EVs were initially described as a mechanism of discarding cellular waste [29], recent studies have demonstrated their crucial role in intercellular communication through active transport of proteins, lipids, and nucleic acids to recipient cells [30]. During viral infections, many viruses exploit the EV machinery to replicate, infect, and evade host immune responses [31,32]. Despite the growing interest in this field, isolation of EVs is still considered an inhibitory factor, as there is no “gold standard”. A number of different approaches have been used to isolate these vesicles, including size exclusion chromatography, ultracentrifugation, precipitation, as well as microfluidics. Since EV biogenesis and viral replication/assembly share common pathways, it is extremely important to choose an isolation procedure in order to minimize the presence of viral particles in EV isolates [33]. For that reason, we adapted a density gradient (iodixanol-based) ultracentrifugation protocol to purify our vesicles. This method provides an effective separation of EVs from viruses with comparable velocities. NTA showed that infection of hcMEC/D3 cells with ZIKV did not increase the size and number of secreted vesicles, possibly indicating that viral particles are not present inside the EVs. This hypothesis was further confirmed by electron microscopy images, where no viral particles were detected in the IEVs. These findings are in agreement with another study by Vora et al. [34], where no intact *DENV* particles (another flavivirus member) were detected in mosquito-cell derived EV fractions. This suggests that our isolation procedure offers good purity. Nevertheless, as previously mentioned, we cannot exclude the possibility that some viral particles might remain in the EV preparations, due to their high similarity and the lack of a universal isolation procedure that removes all viral contaminants. Detection of several EV markers at fractions 3, 4, 5, and 6 in both non-infected and infected conditions clearly indicates that we successfully isolated EVs. In addition, we noticed not only an increased shift in the expression levels of CD63, ALIX, and TSG101, but also the detection of NS1 in fractions 5 and 6 of IEVs. These results could possibly suggest that upper (5 and 6) EV fractions might be used (more often) by the virus to transfer viral material and facilitate further infection. Finally, detection of apolipoprotein A at fractions 5 and 6 clearly indicates that lipoproteins can also co-isolate with EVs at upper fractions, thereby possibly affecting the functional role of EV preparations [35]. 

Furthermore, the presence of both E-protein and viral RNA in our vesicles (even after their treatment with RNase A) suggest that both viral proteins and RNA are highly secured inside EVs, via which they can potentially be transferred to recipient cells. This observation was well-supported by the transwell assay, where hcMEC/D3-cell derived IEVs could pass the initial layer of endothelial cells and deliver their viral content to glial cells, and most probably to other cell types of CNS as well.

Although the general molecular mechanisms by which several viruses, including ZIKV, are able to pass through the tight endothelial layer of BBB have been described [36,37], the exact pathways still remain unknown. Most of these studies focus on permeability, by evaluating transepithelial electrical resistance (TEER) measurements using a voltmeter [38] or by labeling with fluorescein isothiocyanate-dextran [39]. However, the main disadvantages of these two methods are the lack of TEER measurements for the detection of short permeability changes, as well as the inability of TEER to distinguish between resistance and capacitance at different currents [40]. To surpass this limitation, a label-free impedance-based biosensor was applied. This technology allows a real-time measurement of endothelial permeability at multiple frequencies of alternating current (AC) flow and can discern changes in capacitance and resistance. At low AC frequency, the current will mostly flow underneath cells and through the paracellular passage between the cells, while at high AC frequency most of the current will flow capacitively through the cell membranes (transcellular). Hence, changes in impedance at low frequency are more indicative of changes in barrier function while changes in capacitance at high frequency will be indicative of changes in cell monolayer integrity. Interestingly, changes in capacitance could be observed for ZIKV, suggesting that there is a temporary loss in monolayer integrity. The same effect is also observed, but to a lesser extent for IEVs. However, it is extremely difficult to discern the actual mechanism behind this observation, since both values can be affected. Our work provides additional insights to previous studies, leading to the following conclusions. First, inoculation of endothelial cells with ZIKV and IEVs leads to transient disturbances at the early time points, which are restored during the later stages. This illustrates the advantage of using measurements through time, in order to grasp any subtle BBB disturbances [41]. In addition, since the readout gives information on processes that affect paracellular and transcellular currents, we could potentially obtain a better understanding on how BBB works. However, our results are not in agreement with most of the studies that investigate how ZIKV is able to cross the BBB system [38]. This could be explained by the fact that they mainly rely on TEER measurements during the later stages of infection (e.g., 24 h to 48 h p.i.), and not on the first minutes, as we propose [23]. In addition, our analysis was based on the first 2 days of infection. For that reason, we cannot exclude the possibility of BBB disruption at later stages. This disruption could be associated with the secretion of proinflammatory cytokines and chemokines, which are produced during viral infections [42]. To get more insight on what really happens in endothelial cells after EVs or virus entry and to understand what leads to these temporal disturbances, we focused on one of the most abundant adherent proteins of hcMEC/D3, VE-cadherin. This molecule is responsible for controlling the barrier function by regulating the passage of macromolecules in and out of the endothelium [43]. Notably, we observed that the addition of ZIKV and IEVs leads to reorganization of VE-cadherin (temporal changes in the architecture, which leads to gap formation) at 30 min p.i, without affecting its expression levels. However, these alterations were restored after 1 h, possibly indicating that the rapid remodeling of VE-cadherin is one possible mechanism that allows both IEVs and ZIKV to pass through the endothelial cell layer. Although changes in VE-cadherin are mostly related with the paracellular pathway, the concept of interconnectedness between paracellular and transcellular routes leads into a more complicated picture on how VE-cadherin affects the barrier function [44]. However, this needs to be further evaluated. Despite the fact that our findings propose that the observed transient changes of BBB might be correlated with the rearrangement of VE-cadherin, it is extremely important to note that other proteins could also be affected during ZIKV or IEVs entry, including filament actin or the tight junction protein ZO-1 [40]. 

Although the number of EV studies has been rising over the last few years, there are still many unresolved answers regarding their biogenesis and signaling mechanisms. Specifically, it has been described that a number of proteins, such as the endosomal sorting complex required for transport (ESCRT) and tetraspanins, have been involved in these pathways [45,46]. However, new articles have proposed that lipid molecules can also interact with these proteins, and as a result they are likely to be involved in the biogenesis of EVs [47]. In addition, different lipid species have been reported to play a vital role in intracellular signaling [48,49]. To shed more light on this interesting class of macromolecules, we conducted an extensive lipidomic analysis in EV preparations and hcMEC/D3 cells. The most abundant lipid species per class are shown in Figure 5 and Figure S1. In general, our analysis demonstrated that EVs are highly enriched in SM and DG, as compared to the majority of phospholipids. These findings were in accordance with another study [28], indicating their pivotal role in signaling [50]. The relative abundance of several lipid species per class in EVs (as compared to this found in their originating cells) might suggest a selective sorting mechanism during their biogenesis. Interestingly, our study also revealed five lipid species that appeared to be enriched in IEVs, as compared to the NIEVs. From these detected hits, only Cer(d18:0/18:3) was significantly upregulated in the infected condition. Ceramides have been implicated in the biogenesis of EVs, as well in apoptosis [51,52]. Among the other lipid species, PS(18:0/18:1) was found, which is an important phospholipid with multiple functions. Particularly, this phosphatidylserine has been shown to retain cholesterol (role in membrane structure) in the inner leaflet of the membrane, thereby protecting it from oxidation [53]. Furthermore, PS(18:0/18:1) is known to interact and bind with cytoplasmic proteins (e.g., Rab family), thus leading to activation of various signaling cascades [54]. Finally, exposure of PS to the outer leaflet of the membrane has been reported as a recognition marker for apoptosis, which is indicative for viral infections [55]. By summarizing the mechanisms that PS is implicated in, we hypothesize that ZIKV-infected EVs might be able to trigger various signaling pathways via PS(18:0/18:1) upregulation. Regarding the other species, both LacCer and Cer have been involved in intracellular signaling, not only by mediating inflammatory events (secretion of cytokines) but also through production of nitric oxide (NO), during viral infections [22,56]. The fact that Cer(d18:0/18:3) has been found to be significantly upregulated in the IEVs makes this lipid molecule a promising species for future investigation. Further evaluation can promote this ceramide as a potential biomarker during ZIKV infection. 

Finally, we also evaluated the degree of saturation in the majority of lipid classes. Saturated, monounsaturated, diunsaturated, and polyunsaturated acyl chains vary in their structural properties. Interestingly, we found that hcMEC/D3 cells and their secreted vesicles contained much more saturated and monounsaturated acyl chains in most of the detected lipid classes. A high percentage of (mono-un)saturated lipids provides two main advantages to EVs. Firstly, it increases membrane rigidity [57], leading to a more selective sorting mechanism of proteins and lipids into their interior. Secondly, it makes EVs less susceptible to oxidative stress conditions [58]. The latter is of utmost importance during viral infections, since it allows the vesicles to transfer and deliver their content over short and long distances within the extracellular space, without being affected by the presence of reactive oxygen species (ROS). 

To conclude, in this study, we aimed to characterize the potential role of ZIKV-infected EVs (IEVs) on the BBB system. Interestingly, our isolation method revealed the heterogeneity of secreted vesicles, regarding their protein and lipid composition. In addition, we confirmed that IEVs can be used as transport vehicles for viral material, enhancing ZIKV transmission to susceptible cells. For the first time, we showed that both ZIKV and IEVs are able to pass through the BBB and alter monolayer integrity during the early time points of their exposure, possibly by the rearrangement of VE-cadherin. Finally, future studies will be important in order to identify the “key” elements that ZIKV and IEVs use during their entry into BBB.

## Data Availability

All data presented in this study are available within the main text and the Supplemental Material.

## Supplementary Materials

The following are available online at https://www.mdpi.com/article/10.3390/v13122363/s1. Figure S1: Heatmaps representing the lipidomic profile of cell lysates, IEVs- and NIEVs-fractions.
