# Supplementary material for: Deciphering the Role of Extracellular Vesicles Derived from ZIKV-Infected hcMEC/D3 Cells on the Blood–Brain Barrier System
Source: Viruses. 2021 Nov 25;13(12):2363. doi: 10.3390/v13122363 (PMC8708812; doi:10.3390/v13122363)
Supplement: Supplementary file 1 [file viruses-13-02363-s001.zip › viruses-1386065-supplementary.pdf]

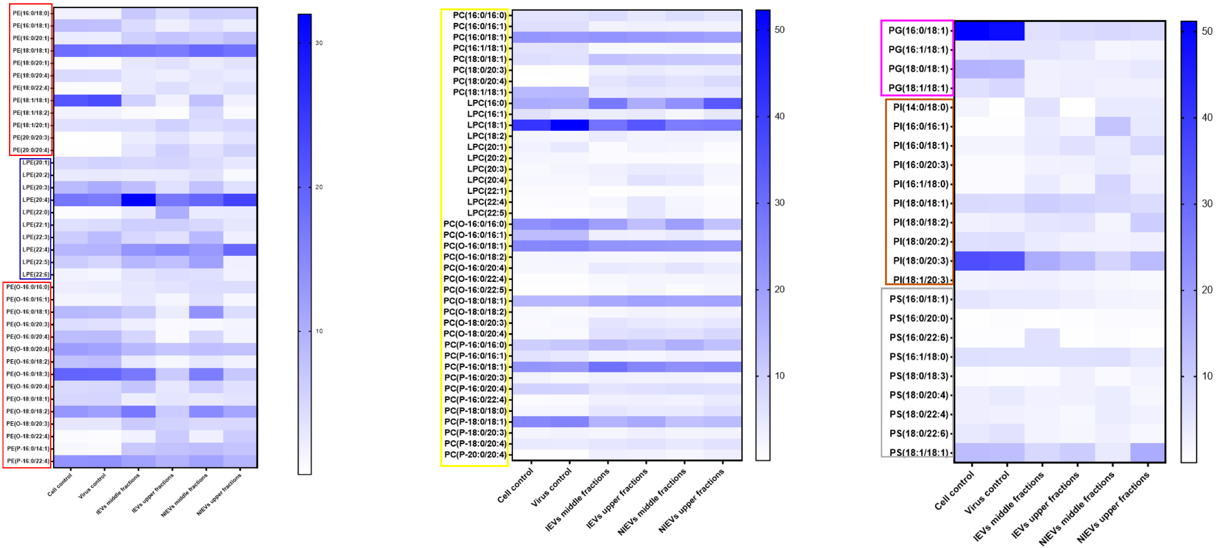

***Supplemental Figure (continued)*: Heatmaps representing the lipidomic profile of cell lysates, IEVs- and NIEVs- fractions.** Several lipid species within each class appear to be enriched in cell lysates, EV preparations or both. Lipid abundance ratios are colored according to their percentage. Higher and lower enrichment are indicated in dark blue and white, respectively. Colored boxes are used to compare the abundance of lipid species within each different class, according to Figure 5A. PE: phosphatidylethanolamine, LPE: lysophosphatidylethanolamine, PC: phosphatidylcholine, LPC: lysophosphatidylcholine, PG: phosphatidylglycerol, PI: phosphatidylinositol, PS: glycerophosphoserine, SM: sphingomyelin, CE: cholesteryl ester, TG: triacylglycerol, DG: diacylglycerol, Cer: ceramide, HexCer: hexosylceramide, LacCer: lactosylceramide. Data shown are from 3 independent experiments.

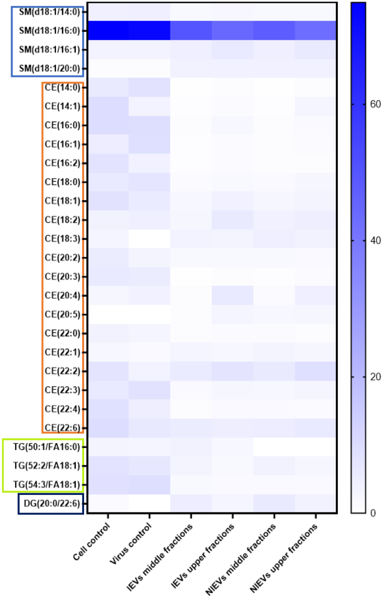
SM(d18:1/14:0)
SM(d18:1/16:0)
SM(d18:1/16:1)
SM(d18:1/20:0)
CE(14:0)
CE(14:1)
CE(16:0)
CE(16:1)
CE(16:2)
CE(18:0)
CE(18:1)
CE(18:2)
CE(18:3)
CE(20:2)
CE(20:3)
CE(20:4)
CE(20:5)
CE(22:0)
CE(22:1)
CE(22:2)
CE(22:3)
CE(22:4)
CE(22:6)
TG(50:1/FA16:0)
TG(52:2/FA18:1)
TG(54:3/FA18:1)
DG(20:0/22:6)
Cell control
Virus control
IEVs middle fractions
IEVs upper fractions
NIEVs middle fractions
NIEVs upper fractions
60
40
20
0

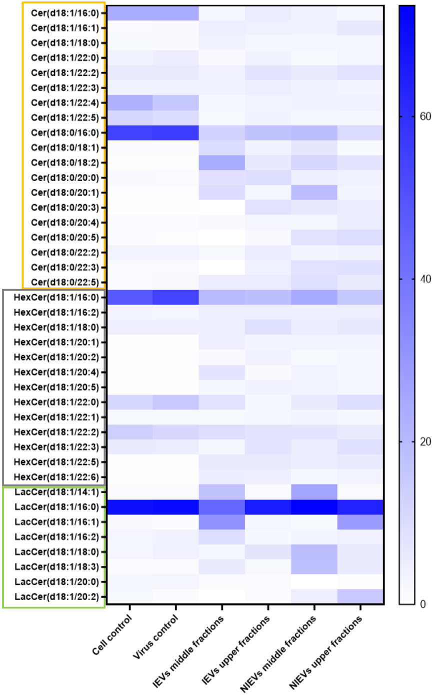
Cer(d18:1/16:0)
Cer(d18:1/16:1)
Cer(d18:1/18:0)
Cer(d18:1/22:0)
Cer(d18:1/22:2)
Cer(d18:1/22:3)
Cer(d18:1/22:4)
Cer(d18:1/22:5)
Cer(d18:0/16:0)
Cer(d18:0/18:1)
Cer(d18:0/18:2)
Cer(d18:0/20:0)
Cer(d18:0/20:1)
Cer(d18:0/20:3)
Cer(d18:0/20:4)
Cer(d18:0/20:5)
Cer(d18:0/22:2)
Cer(d18:0/22:3)
Cer(d18:0/22:5)
HexCer(d18:1/16:0)
HexCer(d18:1/16:2)
HexCer(d18:1/18:0)
HexCer(d18:1/20:1)
HexCer(d18:1/20:2)
HexCer(d18:1/20:4)
HexCer(d18:1/20:5)
HexCer(d18:1/22:0)
HexCer(d18:1/22:1)
HexCer(d18:1/22:2)
HexCer(d18:1/22:3)
HexCer(d18:1/22:5)
HexCer(d18:1/22:6)
LacCer(d18:1/14:1)
LacCer(d18:1/16:0)
LacCer(d18:1/16:1)
LacCer(d18:1/16:2)
LacCer(d18:1/18:0)
LacCer(d18:1/18:3)
LacCer(d18:1/20:0)
LacCer(d18:1/20:2)
Cell control
Virus control
IEVs middle fractions
IEVs upper fractions
NIEVs middle fractions
NIEVs upper fractions
60
40
20
0

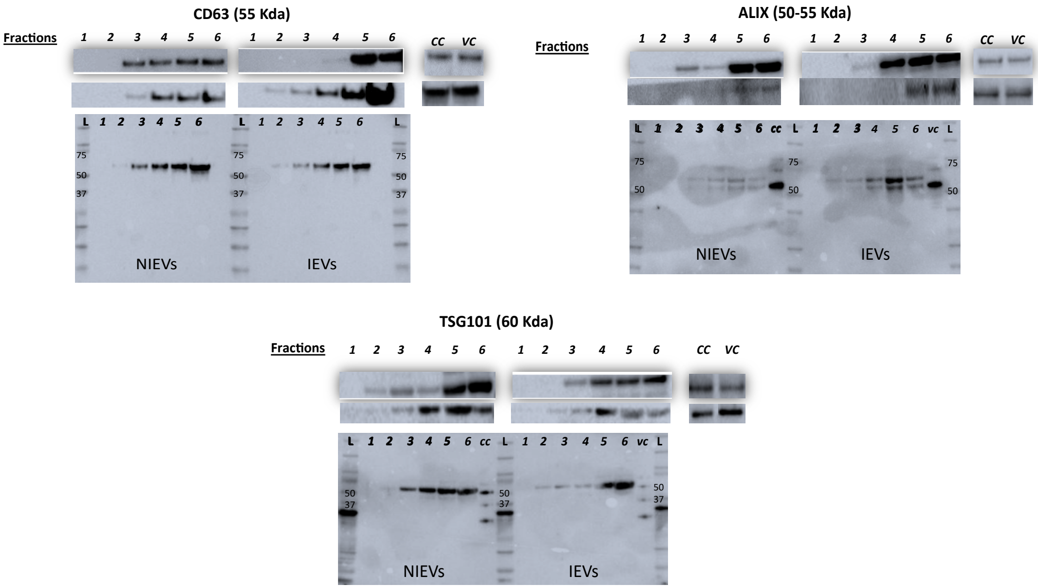
CD63 (55 Kda)
Fractions
1 2 3 4 5 6
1 2 3 4 5 6
CC VC
L 1 2 3 4 5 6 L 1 2 3 4 5 6 L
75
50
37
NIEVs
IEVs
ALIX (50-55 Kda)
Fractions
1 2 3 4 5 6
1 2 3 4 5 6
CC VC
L 1 2 3 4 5 6 cc L 1 2 3 4 5 6 vc L
75
50
NIEVs
IEVs
TSG101 (60 Kda)
Fractions
1 2 3 4 5 6
1 2 3 4 5 6
CC VC
L 1 2 3 4 5 6 cc L 1 2 3 4 5 6 vc L
50
37
NIEVs
IEVs

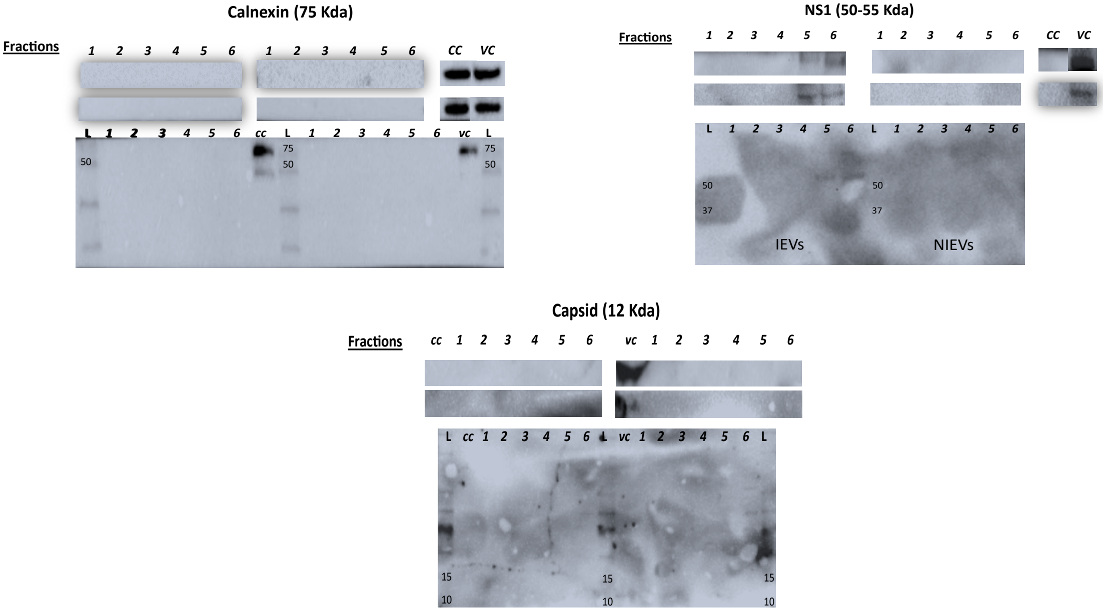
Calnexin (75 Kda)
Fractions
1 2 3 4 5 6 1 2 3 4 5 6 CC VC
L 1 2 3 4 5 6 cc L 1 2 3 4 5 6 vc L
75
50
NS1 (50-55 Kda)
Fractions
1 2 3 4 5 6 1 2 3 4 5 6 CC VC
L 1 2 3 4 5 6 L 1 2 3 4 5 6
50
37
IEVs
NIEVs
Capsid (12 Kda)
Fractions
cc 1 2 3 4 5 6 vc 1 2 3 4 5 6
L cc 1 2 3 4 5 6 L vc 1 2 3 4 5 6 L
15
10
